# Self-Assembly of Cyclodextrin-Coated Nanoparticles: Fabrication of Functional Nanostructures for Sensing and Delivery

**DOI:** 10.3390/molecules28031076

**Published:** 2023-01-20

**Authors:** Busra Cengiz, Tugce Nihal Gevrek, Laura Chambre, Amitav Sanyal

**Affiliations:** 1 Department of Chemistry, Bogazici University, Bebek, 34342 Istanbul, Turkey; 2 Center for Life Science and Technologies, Bogazici University, 34342 Istanbul, Turkey

**Keywords:** cyclodextrin, nanoparticle, host–guest interaction, self-assembly, multivalent interactions, supramolecular chemistry

## Abstract

In recent years, the bottom-up approach has emerged as a powerful tool in the fabrication of functional nanomaterials through the self-assembly of nanoscale building blocks. The cues embedded at the molecular level provide a handle to control and direct the assembly of nano-objects to construct higher-order structures. Molecular recognition among the building blocks can assist their precise positioning in a predetermined manner to yield nano- and microstructures that may be difficult to obtain otherwise. A well-orchestrated combination of top-down fabrication and directed self-assembly-based bottom-up approach enables the realization of functional nanomaterial-based devices. Among the various available molecular recognition-based “host–guest” combinations, cyclodextrin-mediated interactions possess an attractive attribute that the interaction is driven in aqueous environments, such as in biological systems. Over the past decade, cyclodextrin-based specific host–guest interactions have been exploited to design and construct structural and functional nanomaterials based on cyclodextrin-coated metal nanoparticles. The focus of this review is to highlight recent advances in the self-assembly of cyclodextrin-coated metal nanoparticles driven by the specific host–guest interaction.

## 1. Introduction

**Scheme 1:**
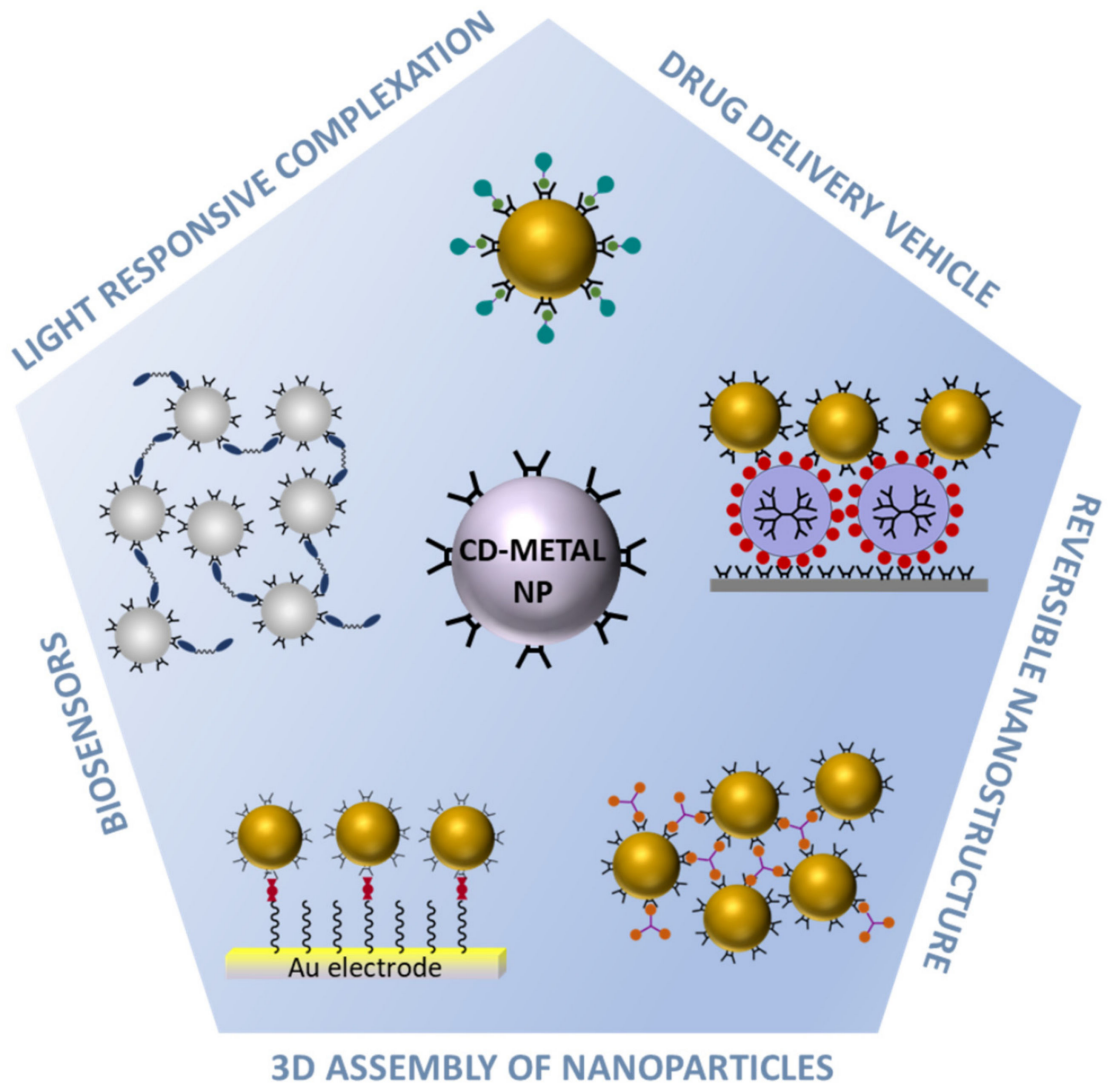
Cyclodextrin-coated metal nanoparticles (CD-METAL NP) and their applications.

Recent years have witnessed an increasing use of metal nanoparticles as building blocks for the fabrication of various nano- and microstructures that find applications in various areas of biomedical and material sciences [1,2]. While discrete nanoparticles in solution are used for several applications such as imaging and sensing, their scope and functional attributes can be enormously broadened through their assembly into clusters or immobilization onto surfaces. Construction of complex and hierarchical structures based on nanoparticles entails the utilization of interactions based on molecular self-assembly that enables them to undergo directed self-assembly to yield desired structures [3,4]. Precise programming of cues at the molecular level allows the nano-building blocks to come together in an orchestrated manner to yield structures that would be otherwise difficult. One or more of the available molecular attractive forces such as electrostatic, dipole–dipole, hydrogen bonding, or specific hydrophobic interactions can be used to elicit interaction between nanoparticles or between nanoparticles and other building blocks of interest. Electrostatic self-assembly utilizing nanoparticles has been extensively employed to generate a myriad of structures using either nanoparticles alone or co-assembled with synthetic molecules and macromolecules or bio-macromolecules such as peptides, proteins, and oligonucleotides [5,6]. Another intermolecular interaction that has been widely employed for the assembly of nanoparticles is based on hydrogen bonding [7]. Hydrogen-bonding-based strategies often benefit from directional and specific interactions that can be achieved by using distinct molecular-recognition dyads. Although widely explored, constructs assembled using hydrogen bonding interactions are not suitable for aqueous environments, which limits their potential usage in many applications. In recent years, the utilization of specific hydrophobic interactions to construct nanoparticle-based assemblies has emerged as a powerful methodology. The approach is becoming increasingly popular and holds immense potential since the assembly is realized under aqueous environments. This is particularly important when functional materials created from nanoparticle building blocks are intended for biological or biomedical applications. Toward this end, the specific interaction between a cyclodextrin-based host and a hydrophobic guest molecule has been utilized to create a variety of nanostructures [8,9]. Cyclodextrins (CDs) are cyclic oligosaccharides composed of six, seven, or eight glucose units and possess a cone-like structure with a relatively hydrophobic (i.e., less hydrophilic) interior cavity and a hydrophilic exterior. The inner cavity provides a suitable binding pocket for hydrophobic guests of appropriate size with high binding association constants in aqueous conditions. This interaction has been employed to design a variety of functional materials from polymeric and inorganic nanomaterials [10,11,12,13,14]. Cyclodextrin-based nano-containers have been immobilized onto various nanoparticle surfaces to provide a handle for specific interaction. Although not addressed in this article, it should be noted that the discrete CD-coated nanoparticles are interesting functional platforms and have been utilized in applications such as chemical sensors, drug delivery, and catalysis [15,16,17,18,19,20] (Scheme 1). The focus of this review is to survey the utilization of CD-coated nanoparticles as building blocks toward the design and construction of various structural and functional self-assembled structures.

## 2. Synthesis of Cyclodextrin-Coated Nanoparticles

**Scheme 2:**
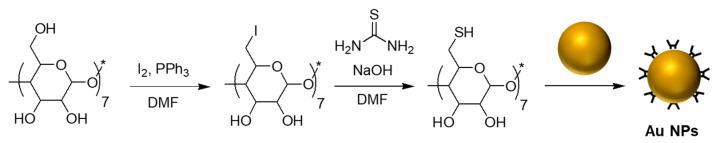
Synthesis of CD-coated gold nanoparticles; DMF: dimethylformamide.

The synthesis of CD-coated metal nanoparticles generally employs appropriately functionalized CD derivatives that have an affinity toward the nanoparticle surface. Commonly used CD-coated nanoparticle systems contain cores that are composed of noble metals such as gold and silver. General procedures involve the synthesis of these noble metal nanoparticles in the presence of thiol-containing CD derivatives. To this end, the primary hydroxy functional groups on a CD are transformed into thiol groups. For example, *per*-6-thio-β-cyclodextrin (β-CD-(SH)_7_) is synthesized from β-cyclodextrin (β-CD) by converting the primary hydroxy groups to iodo groups, followed by their conversion to thiol groups upon treatment with thio-urea [21]. Thiolated-β-CD nanoparticles can be prepared by mixing β-CD-(SH)_7_ with pre-synthesized silver [22] or gold [23] nanoparticles (AgNPs and AuNPs) (Scheme 2). Alternatively, the reduction of gold and silver salts in the presence of perthiolated-CDs/CDs yields CD-coated gold and silver nanoparticles [24,25,26].

Silica nanoparticles coated with CDs can be obtained by attachment of a CD derivative onto an appropriately modified silica nanoparticle. Generally, the surface of silica nanoparticles is coated with nucleophilic functional groups such as amines or thiols. Thus, modified nanoparticles are then treated with CDs containing a toluene-sulfonate moiety, a good leaving group [27]. Alternatively, silica nanoparticles [28] containing carboxylic acid functional groups on their surface can be derivatized by coupling with amine-containing CD molecules. A similar protocol can be used to obtain CD-coated cadmium sulfide (CdS) nanoparticles. Specifically, thioglycolic acid-modified CdS nanoparticles were conjugated with an amine group bearing CD derivative to yield CD-decorated CdS nanoparticles [29]. Additionally, reports disclose that β-CDs are able to bind onto ZnO [30,31,32] or Fe_3_O_4_ [33,34] nanoparticles directly through their hydroxyl groups.

## 3. Self-Assembly of Cyclodextrin-Coated Nanoparticles

In recent years, the “host–guest” type interaction between the CD moiety and hydrophobic guest molecules has been extensively harnessed to direct the self-assembly process of nanoparticles. The cue for such recognition is achieved by the installation of either of the recognition dyads, i.e., the CD moiety or the hydrophobic guest molecule on the nanoparticle surface. Most of the examples reported to date have utilized these interactions to either interface nanoparticles with each other to form clusters or assemble nanoparticles onto planer surfaces or at a liquid–liquid interface. Furthermore, it has been demonstrated that such CD-coated nanoparticles can also be used to assemble with other nanomaterials such as buckyballs and carbon nanotubes that are appropriately functionalized with guest molecules to enable the recognition process. The sections below provide various examples that highlight the crucial role played by the CD moiety on the nanoparticle surface in the construction as well as the function of these systems.

### 3.1. Aggregation of CD-Coated Nanoparticles into Clusters 

**Scheme 3:**
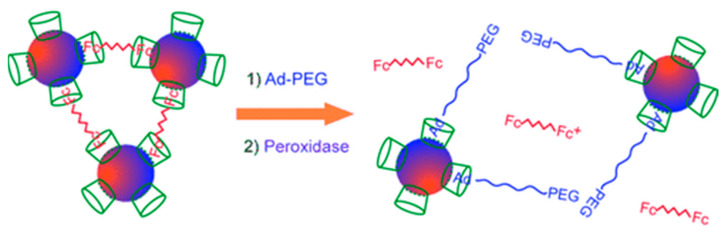
Disassembly of the clusters CD-AuNPs in the presence of horseradish peroxidase and the competing guest molecule; Ad-PEG: adamantyl-functionalized polyethylene glycol, Fc: ferrocene. Adapted with permission from Ref. [38]. Copyright 2011, John Wiley and Sons.

Oftentimes nanoparticles have an inherent tendency to form aggregates and undergo agglomeration, which leads to their insolubility and the decomposition of initially dispersed nanoparticles. The controlled formation of aggregates can be used as a powerful tool to design functional materials since the properties of the aggregates can be quite different from those of individual nanoparticles. For example, while an optical property such as the wavelength of the plasmon band of gold nanoparticles depends on their size, it also depends on the distances between the nanoparticles. Thus, modulation and control of aggregate formation using stimuli such as temperature, pH, solvent polarity, light, redox potential, and specific molecular recognition processes have been investigated. It is known that CDs form stable inclusion complexes with hydrophobic guest molecules such as adamantane (AD) and ferrocene (Fc). Several decades ago, seminal research from Takahashi’s group established that Fc forms a stable inclusion complex with the relatively hydrophobic inner cavity of the CD moiety [35]. While β- and γ-CD form a 1:1 complex, α-CD forms a 2:1 complex with Fc because the smaller size of the α-CDs inner cavity allows partial complexation with the guest molecule. Later research from Evans, Osa, and Kaifer’s group, through a series of electrochemical studies, established that while β-CD binds to the Fc, it does not complex with the oxidized form of Fc [36,37]. In a seminal contribution, Kaifer and coworkers reported the synthesis of gold nanoparticles modified with β-CD and investigated their guest-induced self-assembly. They obtained these receptor-modified nanoparticles by treatment of citrate-capped colloidal gold suspension with β-CD-(SH)_7_. Thereafter, they utilized a Fc-containing dimer to induce the flocculation of these CD-coated nanoparticles. Adjusting the concentration of the Fc-dimer could control the rate of flocculation. Importantly, the study demonstrated that the dynamic nature of this complexation can be exploited to control the flocculation by inducing competitive binding either by the addition of β-CD or a Fc derivative. It was noted that partial dissolution of the flocculates was possible by the addition of excess Fc-methanol [24]. Through a series of voltammetry experiments, it was demonstrated that the CD moiety retains its binding capability toward Fc upon immobilization onto the nanoparticle’s surface. Furthermore, it was shown that the bound Fc could be released by introducing AD, a guest molecule that displays competitive binding with the CD cavity. More recently, using the differential binding of Fc in its reduced and oxidized form, de la Rica, Velders and coworkers, fabricated an ultrasensitive enzyme sensor based on such CD-Fc interactions on AuNPs (Scheme 3). In this work, CD-AuNPs aggregate in the presence of a divalent Fc cross-linker and disassemble by oxidation of Fc unit with horseradish peroxidase (HRP). The sensitivity of detection is highly improved upon oxidation with HRP in the presence of a competitive binder (adamantyl-functionalized polyethylene glycol) [38]. The obtained biosensor has a detection limit as low as approximately 23 HRP molecules without the requirement of further analytic steps.

**Scheme 4:**
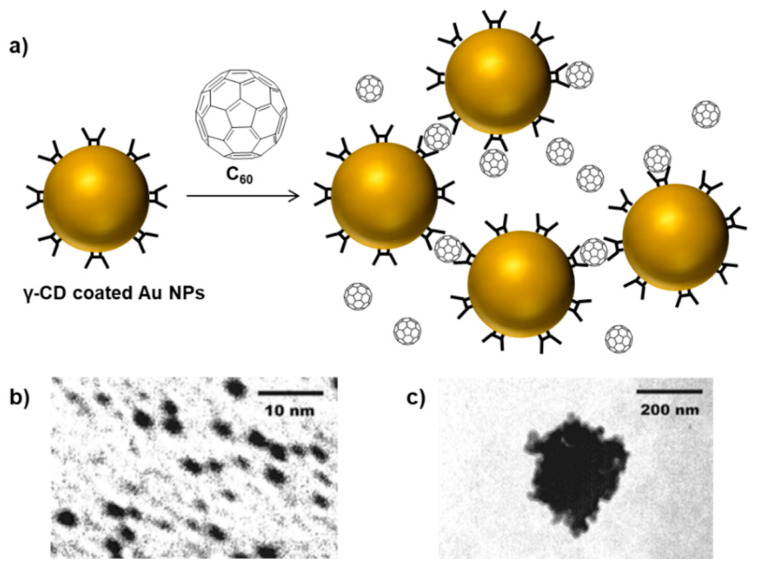
Bridge formation between C_60_ and γ-CD-coated NPs (**a**). TEM images of γ-CD-coated AuNPs (**b**) and C_60_-gold nanoparticle aggregates (**c**). Adapted from Ref. [40]. Copyright 2001, American Chemical Society.

Effects of stimuli such as temperature, pH, biomolecular recognition, and change in solvent polarity have been explored to study the reversibility of such AuNPs aggregates [39]. Similar to the above-mentioned example, it can be envisioned that a large number of divalent guest molecules can be designed to crosslink the CD-coated nanoparticles. Alternatively, the association between a single guest molecule with two CD molecules that results in the formation of a stable complex can also be utilized to produce non-covalent crosslinking. Along these lines, Kaifer and coworkers demonstrated that γ-CD-coated gold nanoparticles upon mixing with C_60_ fullerene molecules result in the formation of aggregates [40]. It was proposed that the C_60_ guest molecule formed a bridge between two γ-CD hosts attached to the surfaces of different nanoparticles (Scheme 4). Photon correlation spectroscopy analysis revealed that the inter-particle non-covalent recognition led to the formation of large network aggregates with an average size of 290 nm according to transmission electron microscopy (TEM) analysis. It was observed that the addition of free γ-CD solution to the C_60_-gold nanoparticle aggregates led to a reversal of aggregation.

**Scheme 5:**
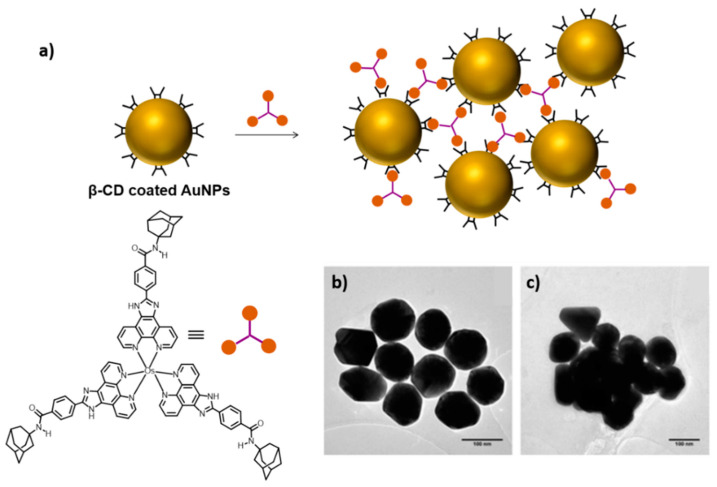
Three-dimensional assembly of CD-modified AuNPs induced by an AD-containing trivalent guest molecule with a redox-active osmium complex at its core (**a**). TEM images of CD-modified AuNPs (**b**) and the host–guest aggregation complex (**c**). Adapted from Ref. [41]. Copyright 2010, American Chemical Society.

Forster and coworkers reported a three-dimensional assembly of CD-modified gold nanoparticles induced by an AD-containing trivalent guest molecule with a redox-active osmium complex at its core (Scheme 5) [41]. Well-defined assembly of AuNPs was achieved due to the rigid octahedral orientation of the three AD guest moieties on the bridging ligand that introduces both photophysical and electrochemical functionality into the assembly. Electrochemical and electron transport properties of multilayers formed on electrode surfaces were investigated by cyclic voltammetry. The homogeneous charge transport rate for the assembly was approximately 40 times higher than for a solid residue of the complex alone, indicating that metal nanoparticles in the network were responsible for the increased charge diffusion.

Jiang and coworkers reported the controlled self-assembly of β-CD-functionalized AuNPs in the presence of a water-soluble ditopic guest molecule with a double-azobenzene structure [42]. The self-assembly process resulted in the shift to higher wavelengths in the absorption spectrum and a visible color change from pinkish red to purple. The aggregation was also verified by TEM. Using UV-Vis spectroscopy and TEM, it was demonstrated that the degree of aggregation can be controlled by adjusting the molar ratio of the host to guest molecules. In this study, the disassembly of the aggregates was accomplished by using the competitive binding of the azobenzene guest molecules with α-CD, another host molecule that has a higher binding affinity. When compared with β-CD, the smaller inner cavity size of α-CD provides stronger hydrophobic interaction with the azobenzene molecule, thus resulting in the formation of a more stable complex.

**Scheme 6:**
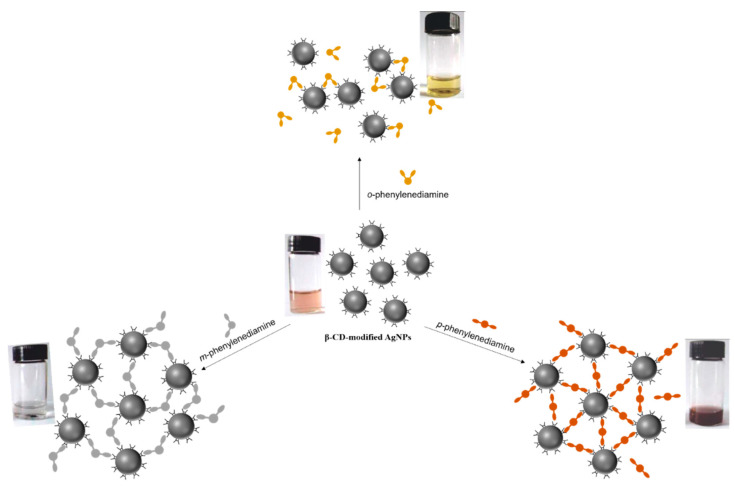
Schematic illustration of host–guest recognition for β-CD-modified AgNPs with different aromatic compounds and photographs of β-CD-modified AgNPs aqueous solution before and after mixing with different isomers of aromatic compounds. Adapted from Ref. [22]. Copyright 2010, American Chemical Society.

The changes in the optical properties resulting from the interaction between the guest molecules and the CD-coated nanoparticles and their assemblies provide an analytical tool to distinguish between the various guest molecules. Along these lines, Zhang and coworkers reported a naked-eye colorimetric detection of different isomers of aromatic compounds, namely dihydroxy- and diamino-phenylenes, using β-CD-coated silver nanoparticles (AgNPs) [22]. The guest molecules or the analytes, although ditopic, were found to present vastly different abilities to simultaneously bind with CD units on two different nanoparticles. Different isomers of the aromatic compounds induced varying extents of aggregations, thus resulting in unique colorimetric responses (Scheme 6). A combination of Coulombic and steric interactions during the formation of the inclusion complexes was indicated to be responsible for the different extent of aggregations.

**Scheme 7:**
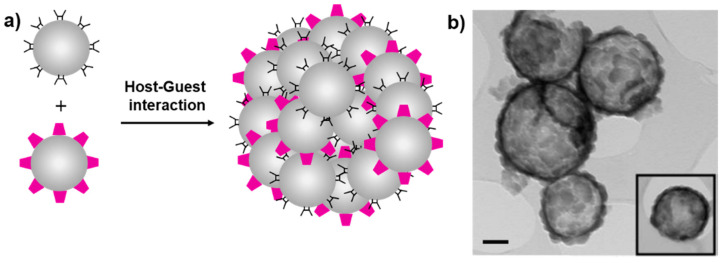
Nanoparticle vesicles through self-assembly of cyclodextrin- and AD-modified silica (**a**) and TEM image of assembled nanoparticles (scale bar 100 nm) (**b**). Adapted from Ref. [43]. Copyright 2010, John Wiley and Sons.

Recently, vesicles have gained increasing interest as scaffolds using supramolecular self-assembly for chemical transportation and storage as well as other spherical nanostructures such as microcapsules and micelles. In 2010, Ritter and coworkers reported the use of nanoparticles to form vesicles by the host–guest interaction of AD- and CD-modified silica nanoparticles [43]. Vesicles were formed and stabilized by hydrophobic host–guest complexation of the adjacent ADs with cyclodextrin groups in aqueous media (Scheme 7). These particles were characterized by dynamic light scattering (DLS) and TEM using their agglomeration and self-assembly behavior in an aqueous solution, while the shell of vesicles was shown by scanning transmission electron microscopy (STEM) images and energy-dispersive X-ray (EDX). Additionally, the dependence of the hydrodynamic diameter of vesicles on the concentration of randomly methylated CD (RAMEB) and the temperature was proven successfully by DLS measurement.

**Scheme 8:**
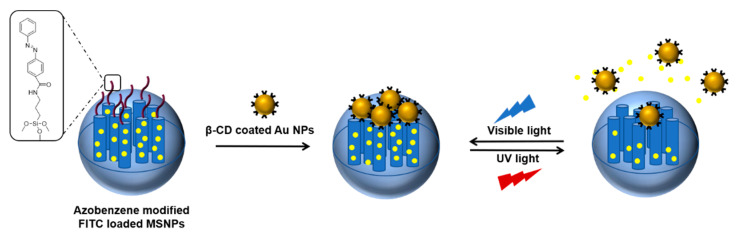
A reversible light-responsive cargo release system based on the host–guest interaction; FITC: fluorescein isothiocyanate, MSNP: mesoporous silica nanoparticles. Adapted with permission from Ref. [45]. Copyright 2018, Royal Society of Chemistry.

The CD-based host–guest interaction can be used to selectively induce interaction between nanoparticles composed of two different materials. In a clever design, Liu and coworkers demonstrated redox-responsive host–guest interaction between ferrocene-coated mesoporous silica nanoparticles (MSNP-Fc) and β-CD-coated AuNPs [44]. In this study, MSNP-Fc serves as a container for fluorescein isothiocyanate (FITC) molecule as cargo, while the β-CD-modified AuNPs attached to MSNP through a host–guest interaction serves as a blocker for controlled release. With the addition of an oxidant, H_2_O_2_, ferrocene oxidation occurs, resulting in its dissociation from the β-CD cavity, and thus releasing the cargo. In a recent paper, the authors showed reversible light-responsive cargo release using the azobenzene/β-CD host–guest interaction [45]. MSNPs were modified with azobenzene and AuNPs were coated with β-CDs. The cargo molecule FITC was loaded into MSNP pores and pores were blocked with β-CD-coated AuNPs (Scheme 8). When UV irradiation is applied, azobenzene groups go into the *cis* configuration and dissociate from the β-CDs, resulting in the release of FITC. On the other hand, with visible light irradiation, azobenzene groups retain the *trans* configuration and regain their association with β-CD.

Another example was studied by Zhao and coworkers which is based on a host–guest interaction between amino β-CDs coated mesoporous silica nanoparticles via cleavable disulfide bonds and PEG-based polymer bearing AD on one end and folate unit on the other end for targeted and controlled delivery of drugs [46]. Doxorubicin was used as a cancer drug and loaded into mesoporous silica nanoparticles. While β-CDs act as a gatekeeper for pores containing drugs, the disulfide bond between MSNPs was utilized for controlled release. Additionally, a folate targeting unit on polymer was used for targeting HeLa cancer cells which are rich in folate receptors. Drug delivery nanoparticles are recognized by folate receptors and enter the cytoplasm, and thus can release doxorubicin via breaking the disulfide bond, which was triggered with a high concentration of glutathione (GSH). In order to check the targeting efficacy, cell experiments were repeated with HEK 293 cells, which are poor in terms of folate receptor and lower efficiency due to the decrease of cellular uptake compared with HeLa cells. 

In another drug delivery work, Enoch and coworkers designed a drug delivery system utilizing host–guest interaction with β-CD attached poly(ethylene) glycol-coated magnetic nanoparticles and the chemotherapeutic agent camptothecin (CPT) [47]. Firstly, one of the lanthanide elements’ erbium-doped nanoparticles was synthesized and they were coated with β-CD attached poly(ethylene) glycol-folate conjugate synthesized earlier. Nanoparticle characterization was done via X-ray diffraction and X-ray photoelectronspectroscopy (XPS). For this nanoparticle carrier, CPT addition resulted in host–guest complex with β-CD with around 88% drug loading percentage. Compared to free drugs, with a nanoparticle carrier system, the efficacy of the drugs and thus the anticancer activity was increased, and the sustained release of drugs was provided.

**Scheme 9:**
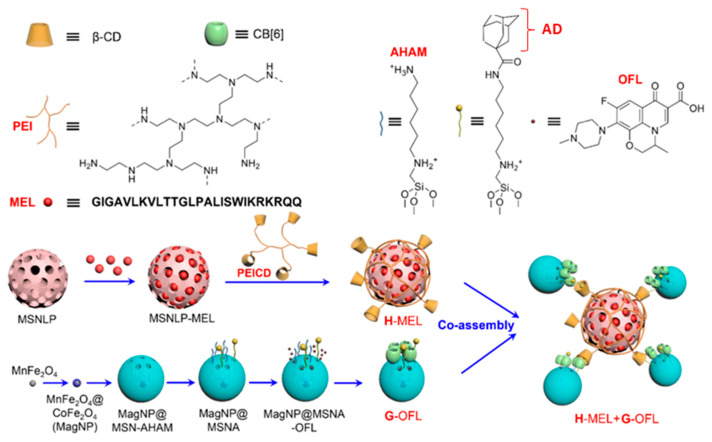
Formation of multi-stimuli-responsive, dual-drug containing mesoporous silica nanoparticle system via the host–guest interaction; PEI: polyethyleneimine, MEL: melamine, OFL: ofloxacin, CB[6]: cucurbit[6]uril, AD: adamantane, AHAM: *N*-(6-*N*-aminohexyl)aminomethyl triethoxysilane, MSNA: Adamantane decorated mesaporous silica nanoparticles. Adapted with permission from Ref. [48] Copyright 2020, American Chemical Society.

Not only drug delivery for cancer treatment but also antibiotic delivery can be achieved using multi-particle systems that are fabricated using a host–guest interaction. A multi-stimuli-responsive model was reported in 2020 by Zink and coworkers [48]. It was mainly based on the interaction between antimicrobial peptide melittin (MEL) loaded MSNPsblocked by β-CD-modified polyethyleneimine (PEI-CD), and antibiotic ofloxacin (OFL)-loaded mesoporous silica nanoparticles containing a magnetic core and capped by cucurbit[6]uril (CB[6]) (Scheme 9). The release of drugs was provided by the stimulus of pathogenic cells and simultaneously heating with an alternating magnetic field (AMF). With the co-delivery of drugs, biofilm eradication was successfully achieved, and it showed much higher potential compared to free drugs and separated host and guest molecules which were inhibited from undergoing multi-particle self-assembly, but were administered simultaneously.

As highlighted in some of the above examples, the non-covalent nature of aggregation allows one to reverse the aggregate formations by introducing competitive binding events. However, for many applications, it would be desirable to tune the state of aggregation without the introduction of other molecules into the system but rather by using an external stimulus such as light, change of pH, or redox potential. It is well known that the azobenzene molecule binds to the β-CD cavity with high affinity when it is in the *trans* form, whereas the *cis* isomer has a lower affinity toward complexation. The *trans*-to-*cis* isomerization of azobenzene can be induced by exposure to UV irradiation. The *cis* isomer reverts back to the *trans* form upon exposure to visible light. Thus, binding between azobenzene and CD moiety is photo-switchable. Ritter and coworkers utilized this photo-responsive binding to induce controlled assembly–disassembly between silica particles [27]. The azobenzene- and β-CD-functionalized SiO_2_ nanoparticles were synthesized from amino-functionalized SiO_2_ nanoparticles which were modified with (phenylazo)benzoic acid and monotosyl-CD, respectively. Photochemically induced aggregation and disaggregation of the nanoparticles were proven by DLS measurements, while the photo-isomerization process was monitored using UV-Vis spectrometry. Importantly, it was deduced that isomerization occurs to a limited extent in the case of inter-nanoparticle complexes and hence full decomplexation does not take place.

**Scheme 10:**
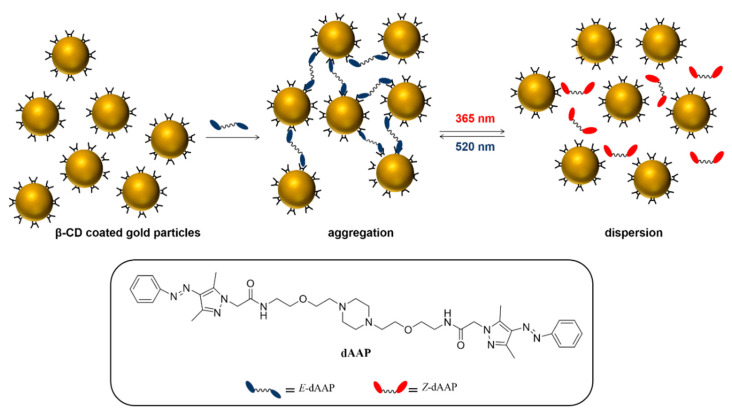
Photo-switchable linking between arylazopyrazoles (AAP) and β-CD-AuNPs. Adapted from Ref. [49]. Copyright 2016, American Chemical Society.

A novel class of molecular switches derived from azobenzenes with enhanced photophysical properties is arylapyrazoles (AAPs), which can also selectively form host–guest complexes with β-CD in the *trans*-configuration. Even though azobenzenes have proven their value in a wide variety of photo-switchable molecular materials, there is a high demand for more efficient light-responsive guest molecules with more stable *Z* isomers. Using a divalent AAP cross-linker, reversible aggregation and disaggregation of gold nanoparticles (AuNPs, diameter ca. 10 nm) functionalized with thiolated β-CD could be achieved [49]. It was shown that the efficiency of the AAP cross-linker in nanoparticle aggregation and disaggregation is higher than for a similar azobenzene cross-linker (Scheme 10). Moreover, disaggregation of the NP clusters could also be induced by near-infrared light excitation and local UV emission of the upconversion nanoparticles (UCNPs), making the system more suitable for applications in biological environments [50].

Kim and coworkers presented a magnetic-field-responsive drug delivery system using cyclodextrin containing polymer-coated super paramagnetic iron oxide nanoparticles (SPIONS) and side chain paclitaxel containing polymers. SPIONs were successively coated with poly[((3-trimethoxysilyl)propyl methacrylate)-r-(PEG methyl ether methacrylate)-r-(*N*-acryloxysuccinimide)] (poly-(TMSMA-r-PEG-r-NAS)) followed by branched PEI (BPEI). Free amino units on SPIONS were then modified by tosylated cyclodextrins and magnetic responsive clusters were obtained by paclitaxel-β-CDs inclusion complex after mixing with pendant paclitaxel-containing polymers. In the presence of an external magnetic field, those clusters exhibited superior antitumor effects against HeLa, MCF-7, and CT26 cancer cell lines and showed remarkable tumor inhibition efficacy in a subcutaneous CT26 tumor model [51].

### 3.2. Assembly of Nanoparticles onto Planar Surfaces

Interfacing discrete nanomaterials with bulk macroscopic materials are oftentimes necessary to design and fabricate functional devices. Directed assembly of nanomaterials onto planar surfaces such as electrodes can be achieved by using a host–guest assembly-based approach. The organization of host or guest molecules onto a planar surface can be used to immobilize nanoparticles decorated with the complementary recognition motif. As an alternative to the direct multivalent interactions between complementarily functionalized nanoparticle surfaces and planar surfaces, one can also utilize multivalent “glue” molecules to bridge the nanoparticles and surfaces decorated with the same recognition motifs.

In an example, Reinhoudt and coworkers reported the aggregation of CD-containing silica nanoparticles and attaching them onto CD print boards by multiple host–guest interactions with guest-functionalized dendrimers [28]. The CD-functionalized silica nanoparticles exhibited aggregation by the addition of the AD-terminated dendrimer, which was able to perform host–guest interaction with CD. According to the change in the average hydrodynamic radius as a function of the concentration of added dendrimer monitored with DLS, aggregation was confirmed. They also proved pH-dependent aggregation by zeta potential measurements in the presence of free amino and carboxylic acid groups on the particle surface. Furthermore, they showed that the same host–guest chemistry was employed to bind the CD-functionalized nanoparticles onto CD print boards on silicon oxide by using AD-terminated dendrimer as molecular glue that attaches CD-modified nanoparticles for strong multivalent host–guest interactions. Thus, it has been shown that certain supramolecular interactions can provide an excellent tool to guide the nanoparticle assembly onto the pre-patterned targeted substrate and control the formation of 2D nanoparticle models.

**Scheme 11:**
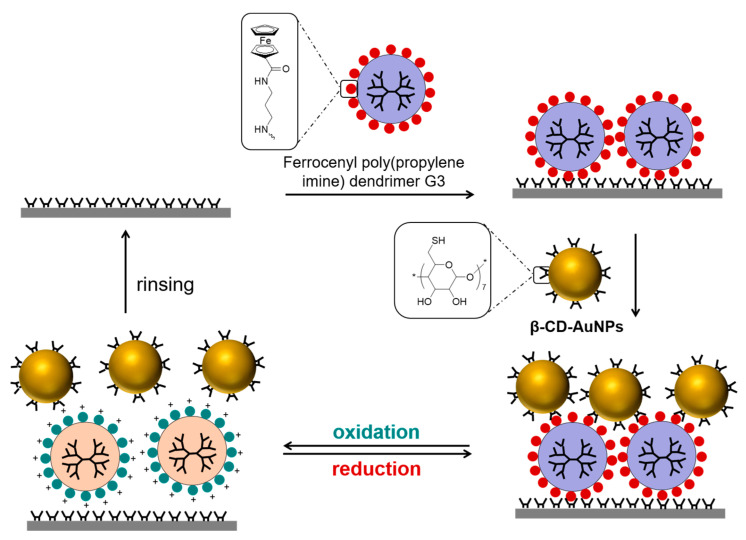
Electrochemically controllable reversible attachment of nanostructure on molecular print boards. Adapted with permission from Ref. [50]. Copyright 2008, American Chemical Society.

Similarly, Huskens et al. focused on the electrochemically controllable reversible attachment of nanostructure at molecular print boards [50]. According to this idea, ferrocenyl-functionalized poly(propylene imine) dendrimers were used as a “molecular glue” between β-CD self-assembled monolayers (β-CD-SAM) and β-cyclodextrin functionalized gold nanoparticles (β-CD-AuNPs) (Scheme 11). It was illustrated that nanoparticles attached to the surface remained stable until electrochemical oxidation of the ferrocenyl end groups led to the desorption of nanostructures from the β-CD-SAM. In a similar way, when electrochemical oxidation was applied to a specific area of a nanoparticle layer, desorption of nanoparticles was observed only in that specific area. To monitor the adsorption and desorption of ferrocenyl dendrimers and β-CD-AuNPs onto and from the molecular print board, surface plasmon resonance spectroscopy (SPR) and an electrochemistry setup were utilized. Repeating the same experiments with β-CD-functionalized silica nanoparticles (β-CD-SiO_2_ NPs) instead of β-CD-AuNPs also proved the reversibility.

**Scheme 12:**
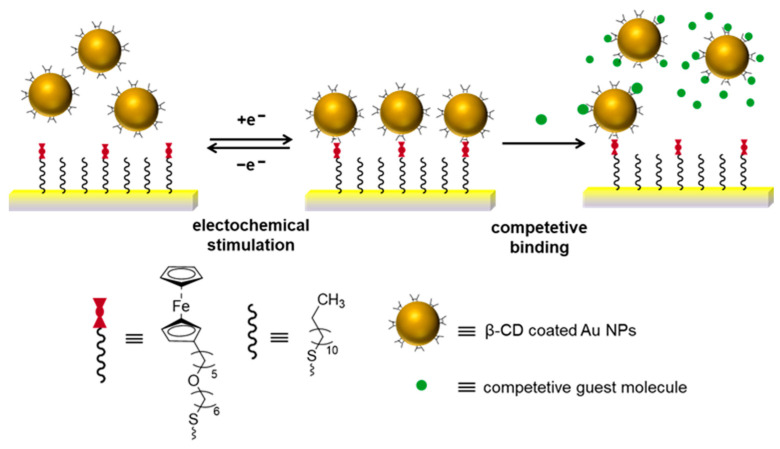
Redox-controlled reversible uptake and release of β-CD-capped AuNPs to and from gold substrate modified with ferrocenyl alkyl thiolates and competitive binding with guest molecules in solution. Adapted from Ref. [52]. Copyright 2012, American Chemical Society.

In a comparable manner, Frasconi and Mazzei reported the electrochemically controlled assembly of β-cyclodextrin functionalized gold NPs (β-CD-AuNPs) on mixed self-assembled monolayers (SAM), involving redox-active ferrocenyl alkyl thiols and n-alkanethiols on gold surfaces (Scheme 12) [52]. Supramolecular complexation or decomplexation between ferrocene moieties and β-CD-capped AuNPs was controlled by the reduction and oxidation of the ferrocene redox probe-modified SAMs and the addition of the competitive guest molecules. Besides the electrochemical stimuli, the light-induced isomerization of azo compounds was employed to direct the binding and release of β-CD-AuNPs to and from the Fc-functionalized surface by using surface plasmon resonance (SPR) spectroscopy. As a result, light- and redox-potential-induced uptake and release of β-CD-AuNPs were achieved.

According to another report published by Harada and Takahashi, β-CD and γ-CD form inclusion complexes with ferrocene and its derivatives [35]. Based on the supramolecular assembly of CDs toward ferrocene, researchers developed a kind of redox-active and supramolecular recognition functionalized electrode (β-CD-AuNPs/Fc-ITO) [53]. To demonstrate this concept, two key components were used. One of them was β-cyclodextrin-capped gold nanoparticles (β-CD-AuNPs), which were used to promote the electron transfer between the analyte and electrode surface and supply the system with the host molecule agent cyclodextrin. The other was ITO (indium tin oxide) coated with a monolayer of ferrocene residues (Fc-ITO). Immobilization of β-CD-AuNPs on the Fc-ITO electrode was confirmed by cyclic voltammetry and atomic force microscopy. By electro-oxidation of the system toward ascorbic acid, the electrocatalytic activity of the β-CD-AuNPs/Fc-ITO electrode, whose assembly is driven by supramolecular interactions, was proven according to the data obtained from cyclic voltammetry.

**Scheme 13:**
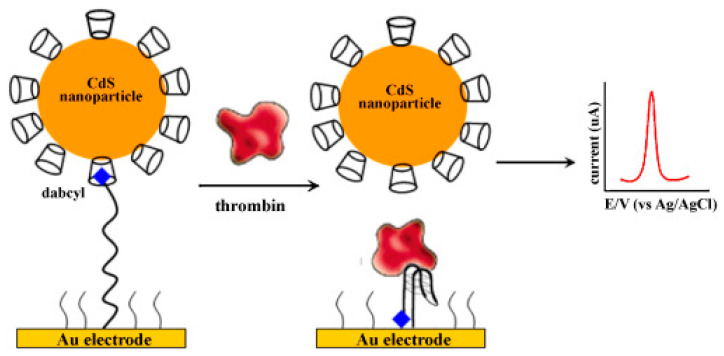
Schematic of the assembly of the electrochemical aptasensor for the detection of thrombin. Reprinted with permission from Ref. [54]. Copyright 2012, Elsevier.

Fang and coworkers reported the construction of an electrochemical aptasensor for thrombin detection based on a host–guest molecular recognition. In this work, using a dabcyl- and thiol-labeled thrombin-binding aptamer (TBA), a single-stranded 15-mer DNA was immobilized on an Au electrode surface via Au-S bond and used as a probe for sensing the target thrombin protein [54]. This way, CDs coated on CdS nanoparticles act as both electrochemical signal providers and convenient hosts for the dabcyl guest. To fabricate the aptasensor, the host–guest interaction between CdS nanoparticle surface-modified β-cyclodextrins (CdS-CDs) and dabcyl-labeled TBA-modified electrode was used. Following the aptasensor fabrication by the host–guest interaction between cyclodextrin and dabcyl units, thrombin protein captured onto the aptasensor electrode surface due to the specific binding of aptamer toward thrombin which led to the removal of CdS-CDs from the electrode surface (Scheme 13). Since released CdS nanoparticles offered an electrochemical current signal, thrombin detection was proven. By comparing previous biosensor methods, the authors pointed out that this approach shows efficient detection and sensitivity.

In a more recent work, Fan and coworkers developed a functionalized nanocomposite-based electrochemiluminescence (ECL) sensor for detecting thrombin [55]. In this study, Ru(bpy)_3_^2+^/β-CD-AuNPs/nanographene (NGP) composites were used to modify the glassy carbon electrode (GCE) surface, and then aptamers (TBA1 and TBA2 with a 1:1 M ratio) were labeled with Fc to act as the probed and were attached to the composites via the host–guest recognition between β-CD and Fc. In the absence of thrombin, the quenching of Fc to [Ru(bpy)_3_]^2+^ was maintained, and “signal-off” ECL was observed. However, because of the specific combination of the aptamer probes and thrombin, the configuration of aptamer probes changed and escaped from the electrode surface once thrombin appears, which results in the quenching disappearance, and the ECL signal was changed from “off” to “on”.

**Scheme 14:**
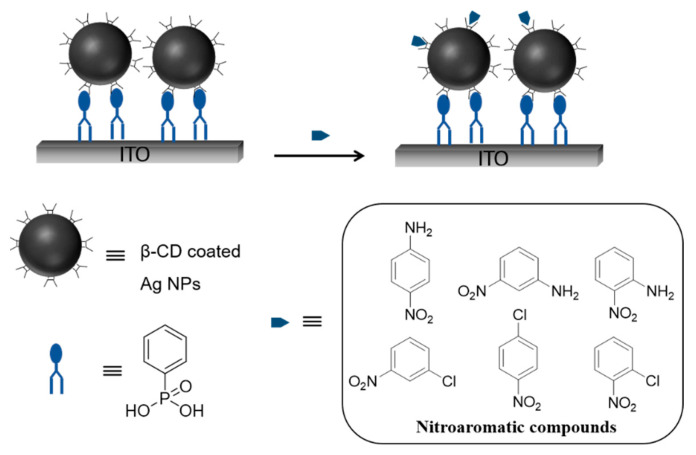
Attachment of β-CD-coated Ag NPs to phenylphosphonic acid immobilized on indium tin oxide (ITO) electrode through the host–guest interaction between the β-CD and the phenyl ring of the phenylphosphonic acid. Adapted from Ref. [67]. Copyright 2012, American Chemical Society.

The recent attention being paid to electrochemical sensors has let the investigation of different types of materials being used in sensors. Among these, nanomaterials have preferable advantages of stability, sensitivity, and selectivity. Especially in designing sensors for required properties, metal nanoparticles are crucial to achieving selective determination of specific compounds [56,57,58,59,60,61,62]. Some studies have demonstrated that AgNPs are suitable for electron transfer ability and electrocatalytic reduction of various molecules [63,64,65,66]. One of the important sensor studies is based on the detection of nitroaromatic compounds prepared by β-CD modified silver nanoparticles (AgNPs) [67]. In this work, the sensor design was based on the idea of inclusion complexation of nitroaromatic isomer guests with the β-CD host. For such an aim, β-CD-coated AgNPs were attached to phenylphosphonic acid immobilized indium tin oxide (ITO) electrode through the host–guest interaction between the β-CD and the phenyl ring of the phenylphosphonic acid (Scheme 14). Available β-CD hosts were utilized for sensing different nitroaromatic compounds. Moreover, isomers were identified on account of their different binding strengths to the β-CD host proven by square wave voltammetry (SWV) and cyclic voltammetry (CV) results.

**Scheme 15:**
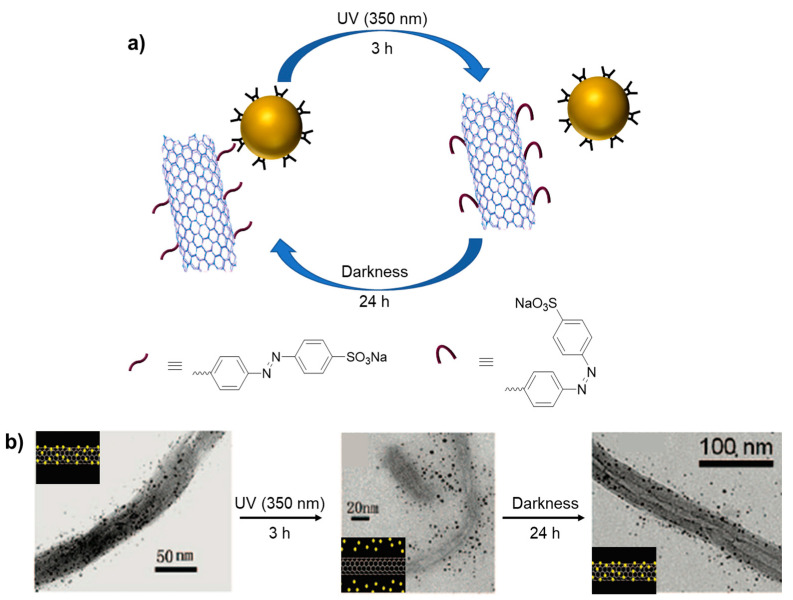
Photo-reversible mechanism from host–guest interaction between α-CD-coated gold nanoparticles and multi-walled carbon nanotubes (**a**) and TEM images of reversible attachment (**b**). Adapted with permission from Ref. [73]. Copyright 2009, American Chemical Society.

Another important approach based on nanoparticle self-assembly is supramolecular layer-by-layer (LbL) systems. In 2005, Huskens and coworkers published a report on multivalent host–guest interactions between AD-functionalized dendrimers and CD-coated gold nanoparticles [68]. Since the synthesized generation-5 AD-terminated poly-(propylene imine) (PPI) dendrimer (with 64 AD groups) is not water-soluble, to achieve the combination of dendrimers with cyclodextrin self-assembled monolayers on a gold substrate (CD SAMs) and cyclodextrin modified gold nanoparticles (CD-AuNPs), AD end groups were complexed with slightly excess CD and the core amine groups of dendrimers were protonated (Scheme 15). The combination of their aqueous solutions at a certain pH resulted in the fabrication of multilayer supramolecular self-assembly characterized by UV-Vis absorption spectroscopy, ellipsometry, and atomic force microscopy (AFM).

In a similar way, Huskens and coworkers revealed another supramolecular LbL system using nanoimprint lithography for the confinement of the nanostructures [69]. Nanoparticles with different sizes and core materials were prepared to create the combination of a multilayered hybrid system comprising organic, metallic, and inorganic nanostructures which were CD-functionalized silica nanoparticles (CD-SiO_2_, 350 nm), ferrocenyl-functionalized silica nanoparticles (Fc-SiO_2_, 60 nm) and CD-functionalized Au (CD-Au, 3 nm), respectively. Each nanoparticle took part in specific adsorption onto CD-mobilized silicon oxide surfaces (CD SAMs) by alternating assembly through their host–guest recognition units. Additionally, generation 1-adamantyl-functionalized poly(propyleneimine) dendrimers (G1-PPI-(AD)4) were used as a molecular glue for absorption of cyclodextrin-coated nanoparticles onto CD SAMs via host–guest recognition technique. Researchers focused on the comparison of the impacts of the sequence of the nanoparticle assembly steps, from large to small and from small to large nanoparticles, which were observed by AFM imaging.

In another study, Yue and coworkers demonstrated an “ON-OFF-ON” sensor that uses competitive host–guest interaction for melamine (MEL) detection in milk [70]. β-CD-modified carbon nanoparticles (β-CD-CNPs) were synthesized as host nanoparticles and both Fe^3+^ and MEL in milk were used as two competitive guest molecules. While β-CD provides a relatively hydrophobic cavity for Fe^3+^ and MEL, fluorophore CNPs provide excellent fluorescence properties to β-CD-CNPs (ON). Thus, When Fe^3+^ is presented to the medium, it interacts with the β-CD cavity and makes a host–guest complex which causes fluorescence quenching of the system (OFF). On the other hand, when MEL enters the medium, it competes with Fe^3+^ and makes a stronger host–guest complex with β-CD. Finally, Fe^3+^ is removed from the β-CD cavity while MEL takes its place, and the fluorescence of the system switches ON. The sensor was applied to an analysis of MEL in milk samples, and it gave a high level of sensitivity and selectivity in MEL detection in these real-life samples.

In addition to the planar surface, the assembly of nanoparticles by host–guest interaction was shown on multi-walled carbon nanotubes (MWCNTs). The valuable electrical, thermal, and optical properties of carbon nanotubes and their exceptionally high mechanical strength and stability have made them suitable for many applications [71,72]. Liu and coworkers described a photo-reversible host–guest interaction between cyclodextrin-coated gold nanoparticles and azobenzene-coated multi-walled carbon nanotubes (MWCNTs) [73]. As it is known, α-cyclodextrin (α-CD) forms inclusion complexation with the trans form of azobenzene derivatives which turns into *cis* form under UV irradiation at 360 nm (Scheme 15). Due to the change in molecular volume of azobenzene moiety, the CD can no longer include this bulky *cis* form of azobenzene, resulting in the exclusion of a supramolecular system [74,75]. Leveraging the photoisomerization property of azobenzene, the α-CD (host) coated gold nanoparticles formed photochemically controlled inclusion–exclusion interaction with the azobenzene (guest) derivative, which was covalently bonded onto the surface of MWNTs. The reversible attachment of gold nanoparticles onto the MWNT surface was demonstrated by Fourier-transform infrared spectroscopy (FTIR), TEM, Raman spectroscopy, and thermal gravimetric analysis (TGA).

### 3.3. Assembly of Nanoparticles at Liquid–Liquid Interfaces

**Scheme 16:**
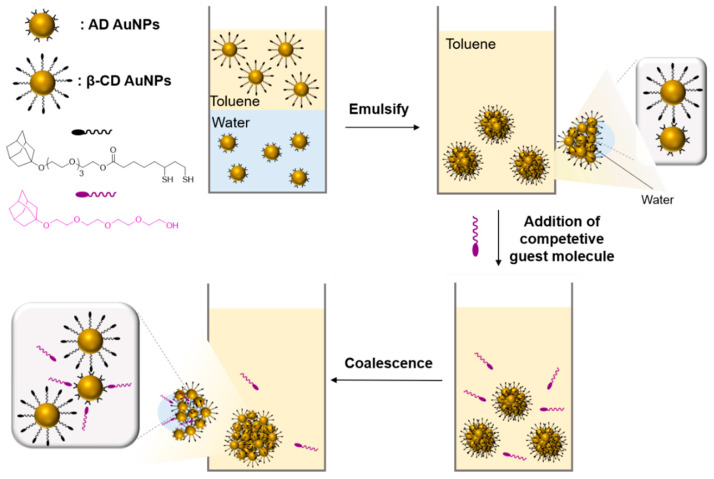
Formation and size tuning of colloidal microcapsules via host–guest molecular recognition at the liquid–liquid interface. Adapted with permission from Ref. [80]. Copyright 2009, American Chemical Society.

The field of host–guest chemistry in materials science has attracted great attention since this promising method allows the development of different scaffolds enabling multivalent interactions, one of which is colloidal microcapsules (MCs). Among the number of examples for the colloidal microcapsules [76,77,78,79], the work reported by Rotello and Sanyal used host–guest chemistry to prepare well-defined stimuli-responsive colloidal microcapsules [80]. MCs were fabricated by crosslinking between water-soluble β-cyclodextrin-coated gold NPs (β-CD-AuNPs) and organo-soluble AD functionalized gold NPs (AD AuNPs) at the oil–water interface as a result of vigorous shaking of nanoparticle solutions (Scheme 16). After the preparation of emulsions, MCs remained stable due to multivalent interactions of multiple complementary ligands on the nanoparticle surface. Since MCs were obtained with non-covalent hydrophobic interactions, they had a reversible nature. Therefore, the size of these MCs was tunable via the introduction of competing AD-containing amphiphilic guest molecules. The size tunability of the colloidal MCs was proven both by optical and fluorescence microscopes.

In a following study, Rotello, Sanyal, Crosby, and coworkers reported the fabrication of buckled microparticles via host–guest interactions of 1-adamantoyl-6-(4-phenyl)azobenzoyl-hexane (AB-Hex-AD), 1,6-di(4-(phenyldiazenyl)benzoyl)-hexane (AB-Hex-AB), and 1,6-diadamantoylhexane (AD-Hex-AD) linkers dissolved in chloroform and β-CD-coated AuNPs in water. Divalent molecular guest linkers crosslinked the interfacial nanoparticles to generate stable microcapsules with a robust nanoparticle membrane shell. The inner oil phase containing dicyclopentadiene and catalysts was polymerized to obtain stable microparticles with measurable buckled surfaces. Interestingly, the mechanical strength of the outer membrane was observed to depend on the binding affinities between the host NPs and the guest linkers [81].

**Scheme 17:**
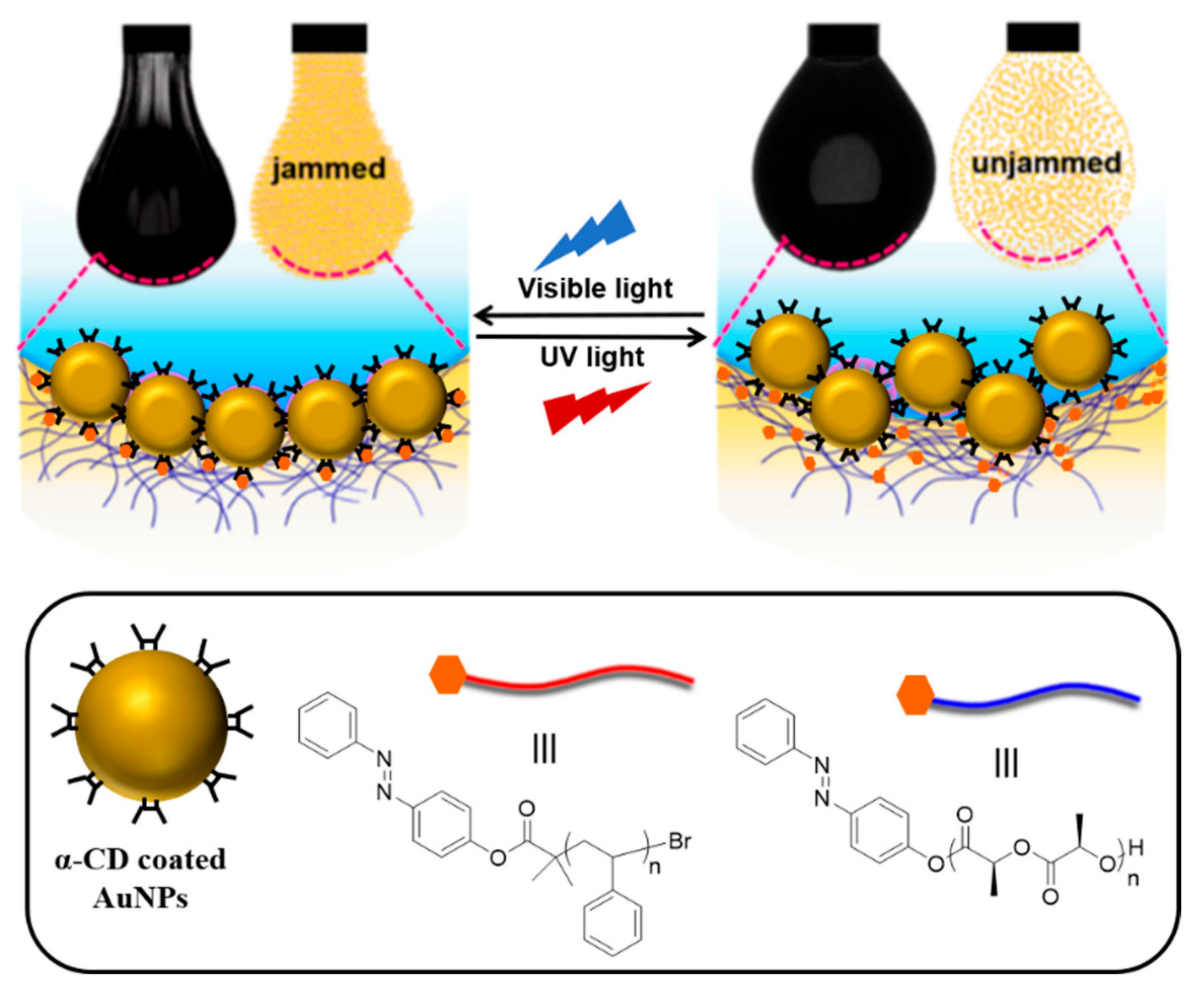
Photo-responsive NPS at the oil–water interface switching from the jammed state to the unjammed state reversibly. Adapted with permission from Ref. [82]. Copyright 2020, American Chemical Society.

More recently, Shi and coworkers designed a photo-responsive nanoparticle surfactant (NPS) [82] which was constructed with the host–guest interaction at the oil–water interface between water-soluble α-CD-coated AuNPs (diameter ca. 10 nm) and oil-soluble polymeric structures which are azo-terminated polystyrene (Azo-Ps) and azo-terminated poly-L-lactide (Azo-PLLA) (Scheme 17). Azobenzene in the *trans* form can interact with CD under visible light, while after irradiating with UV azobenzene turns into the *cis* form and can no longer interact with CD. By using this photo-controlled molecular recognition feature, reversible jammed (solid-like droplets) and unjammed (liquid-like droplets) states of NPS were generated. In terms of easy manipulation in changing of shapes and dispersion states of droplets, the designed NPS can be utilized for delivery in fluid systems. In a subsequent report, Shi, Russell, and coworkers proposed a supramolecular redox-responsive system utilizing host–guest interaction of β-CD and ferrocene (Fc) at the toluene–water interface [83]. In the biphasic system including β-CD-coated gold nanoparticles and ferrocene-terminated poly-l-lactic acid (Fc-PLLA), by using the assembly of reduced Fc with β-CD via NaClO and disassembly of oxidized Fc via Na_2_S_2_O_4_, reversible encapsulation of cargo (Rhodamine B) and its release were achieved. As an impressive demonstration, the authors were able to 3D print dye-encapsulated tubules of water in toluene using the liquid structuring ability of the supramolecular colloidal assembly at the interface and release the encapsulated cargo through disassembly of the interface by changing the redox state of Fc.

## 4. Conclusions

In this review, recent advances in the self-assembly of cyclodextrin-coated nanoparticles driven by the specific host–guest interactions have been summarized. In the past decade, the bottom-up approach has arisen as a powerful tool in nanofabrication for the field of materials and biomedical sciences. Such an approach for the fabrication of functional materials requires the utilization of specific molecular interactions. In this regard, the cyclodextrin-mediated interactions embody several attractive features such as high specificity, stimuli-responsive association, and dissociation, but, most importantly, the assembly takes place in an aqueous environment. The latter attribute enables engineering functional constructs appropriate for many biomedical applications spanning from disease diagnostics to drug delivery. One can see from the present review the several attractive features of cyclodextrin-coated nanoparticles that serve to excite and fire the imagination of new and established researchers to utilize these fascinating building blocks to create new functional materials for addressing global challenges in the area of biomedical sciences.

## Data Availability

Not applicable.
